# Person Re-Identification with RGB-D Camera in Top-View Configuration through Multiple Nearest Neighbor Classifiers and Neighborhood Component Features Selection

**DOI:** 10.3390/s18103471

**Published:** 2018-10-15

**Authors:** Marina Paolanti, Luca Romeo, Daniele Liciotti, Rocco Pietrini, Annalisa Cenci, Emanuele Frontoni, Primo Zingaretti

**Affiliations:** Department of Information Engineering, Universitá Politecnica delle Marche, I-60131 Ancona, Italy; l.romeo@univpm.it (L.R.); d.liciotti@pm.univpm.it (D.L.); r.pietrini@pm.univpm.it (R.P.); a.cenci@pm.univpm.it (A.C.); e.frontoni@univpm.it (E.F.); p.zingaretti@univpm.it (P.Z.)

**Keywords:** RGB-D camera, person re-identification, machine learning, K-nearest neighbors, retail

## Abstract

Person re-identification is an important topic in retail, scene monitoring, human-computer interaction, people counting, ambient assisted living and many other application fields. A dataset for person re-identification TVPR (Top View Person Re-Identification) based on a number of significant features derived from both depth and color images has been previously built. This dataset uses an RGB-D camera in a top-view configuration to extract anthropometric features for the recognition of people in view of the camera, reducing the problem of occlusions while being privacy preserving. In this paper, we introduce a machine learning method for person re-identification using the TVPR dataset. In particular, we propose the combination of multiple k-nearest neighbor classifiers based on different distance functions and feature subsets derived from depth and color images. Moreover, the neighborhood component feature selection is used to learn the depth features’ weighting vector by minimizing the leave-one-out regularized training error. The classification process is performed by selecting the first passage under the camera for training and using the others as the testing set. Experimental results show that the proposed methodology outperforms standard supervised classifiers widely used for the re-identification task. This improvement encourages the application of this approach in the retail context in order to improve retail analytics, customer service and shopping space management.

## 1. Introduction

Camera installations are widespread in several domains, from small business and large retail applications, to home surveillance applications, environment monitoring, facility access, sports venues and mass-transit. Identification cameras are widely employed in most public places like malls, office buildings, airports, stations and museums. In these applications, it is desirable to identify different instances or images of the same person, recorded at different moments, as belonging to the same subject. This kind of process, commonly known as “person re-identification” (re-id), has a wide range of applications and is of great commercial value.

Research in people behavior analysis has been thoroughly focused on person re-id during the last decade, which has seen the exploitation of many paradigms and approaches of pattern recognition [1,2,3]. In challenging situations, algorithms need to be robust to be able to deal with issues such as widely-varying camera viewpoints and orientations, rapid changes in the appearance of clothing, occlusions, varying poses and various lighting conditions [4,5].

The first studied re-id problem was related to vehicle tracking and traffic analysis, where objects move in well-defined paths, have almost uniform colors and are rigid. Features like color, speed, size and lane position are generally embedded in Bayesian frameworks. However, person re-id requires more elaborate methods in order to deal with the widely-varying degrees of freedom of a person’s appearance [6].

Much of the research on person re-id has been devoted to modeling human appearance. In fact, descriptors of image content have been proposed in order to discriminate identities while compensating for appearance variability due to changes in illumination, pose and camera viewpoint. Re-id is also a learning problem in which either metrics or discriminative models are actually learned [5,7]. Labeled training data are required for metric learning approaches, and new training data are needed whenever a camera setting changes [8].

Recently, person re-id has emerged as a very interesting tool for detection and tracking of people under occlusion or partial camera coverage. In a retail environment, re-id can provide useful information for improving customer service and shopping space management. In fact, changes in consumer purchase behavior led retailers to adapt their businesses, the products and services provided, as well as the way they communicate with customers. In the retail field, person re-id becomes a useful tool to recognize consumers in a store properly, to study returning consumers and to classify different shopper clusters and targets. The customer interactions such as (i) the level of attraction (i.e., attraction that the shelf is creating for consumers), (ii) the attention (i.e, the time consumers spend in front of a brand display) and (iii) the action (i.e., the number of consumers that enter the store and interact with particular merchandise) can be closely monitored through RGB-D cameras. This solution provides affordable and additional rough depth information coupled with visual images, offering sufficient accuracy and resolution for indoor applications. A distributed RGB-D camera has already been successfully applied in the retail field to identify customers univocally and to analyze behaviors and interactions with shoppers [9,10]. The choice of the RGB-D camera in a top-view configuration is preferred due to its greater suitability compared with a front view configuration, usually adopted for gesture recognition or even for video gaming. The top-view configuration reduces the problem of occlusions and has the advantage of being privacy preserving because a person’s face is not recorded by the camera [11]. Top-view people counting applications are the most accurate (with accuracy up to 99%) even in very crowded scenarios (more than three people per square meter) [12]. The point of view of the camera in the top-view configuration is also the only one that allows measuring anthropometric features of the people passing by and interactions among shoppers and products on the shelf at the same time [13,14]. However, this configuration may lead to an important limitation: it does not allow one to retrieve features related to the front view that are widely employed in other state-of-the-art approaches (e.g., [15,16]), in which the subject identification can be highly discriminative. Hence, the proposed approach including the feature extraction and the classification stage was designed according to this challenging setup.

Currently, several datasets using RGB-D technology are available for the study of person re-id and cover many aspects of this problem, such as shape deformation, occlusions, illumination changes, very low resolution images and image blurring [17]. The most popular are VIPeR [18], the iLIDSmulti-camera tracking scenario [19], ETHZ [20], CAVIAR4REID [21] and [22]. However, since these datasets are not collected in a top-view configuration, they are not suitable for our purposes.

In this regard, we have built a new dataset for person re-id that uses an RGB-D camera in a top-view configuration: the TVPR (Top View Person Re-identification) dataset [23], using an Asus Xtion Pro Live RGB-D camera, which allows the acquisition of color and depth information in an affordable and fast way [24]. The camera was installed on the ceiling above the area to be analyzed. This dataset includes the data of 100 people, acquired across intervals of days and at different times.

Differently from [23], the main goal of the paper comprises the introduction of the feature extraction and classification stage for the re-id task in a top-view configuration scenario using a set of features extracted by the color and depth images. The overall system comprises the recording stage, the pre-processing/feature extraction stage and the classification stage. Thus, we have tested the approach using the TVPR dataset [23] with respect to other state-of-the-art classifiers in order to measure the reliability and the effectiveness of our approach. In particular, we propose an ensemble method, named Multiple K-Nearest Neighbor (MKNN), based on the combination of different k-Nearest Neighbor (K-NN) classifiers. The problem of combining different K-NN has been addressed in [25,26,27] respectively for different feature subsets and different distance functions. The main contributions of this work with respect to the existing literature are: (i) the adoption of different distance functions for each single K-NN based on the nature of the feature descriptors, (ii) the introduction of Neighborhood Component Feature Selection (NCFS) for the anthropometric features, (iii) the overall combination method and (iv) the application of the following methodology on the TVPR dataset collected by the authors in a previous work [23]. The motivation for the usage of the specific method, i.e., MKNN, arose from the need to exploit the informative power of depth and RGB input properly combining the different nature of each feature. Although the authors combined different existing classifiers in an ensemble strategy, the way these classifiers were chosen and combined represents the main advantage of the proposed classification stage. The experimental results demonstrated the effectiveness of the proposed approach, encouraging its application in public contexts and in different real-world applications (e.g., safety and security in crowded environments, access control), where the top-view configuration allows reducing the problem of occlusions and privacy.

Each K-NN is trained by different distance functions and feature subsets. The neighborhood component feature selection is applied to the depth features to find the optimal weights, while cosine distance and Spearman’s rank correlation are applied to measure the similarity between two RGB feature points. Instead of the standard majority vote method, we propose a variation of the Bayesian approach for combining the decision of different K-NN. The performance evaluation encourages the reliability and the effectiveness of the proposed approach. The MKNN methodology decreases the generalization error compared to the baseline K-NN method, outperforming supervised classifiers used for the re-id task (i.e., K-Nearest Neighbors (K-NN) [28], Decision Tree (DT) [29] and Random Forest (RF) [30,31]).

The paper is organized as follows: Section 2 provides a description of the approaches in the context of re-id (Section 2.1) and the characterization of the TVPR dataset (Section 2.2). Section 3 gives details on the proposed methodology for the feature extraction stage and the machine learning model implemented. Section 4 provides the experimental results and comparison with respect to baseline classifiers. The conclusions and future work in this direction are proposed in Section 5.

## 2. Background

This section presents an overview of the main approaches in the context of person re-id. In particular, Section 2.1 provides a review/summary of the literature on person re-id methods, and Section 2.2 gives details on the TVPR dataset for person re-id in a top-view configuration.

### 2.1. Previous Works on Person Re-Identification

Over the past few years, in the field of object recognition, the re-id problem has received considerable attention, and various reviews and surveys are available, pointing out different aspects of this topic [32,33]. Among the proposed approaches, four different classes could be defined, mainly depending on the camera setup and environmental conditions: biometric, geometric, appearance-based and learning approaches.

In the biometric approaches, the different person instances are matched together and are assigned to the same identity by the use of biometric features. The examples adopted in the real situation involve gait, faces, fingerprints, iris scans, and so on [34,35]. They are reliable and effective solutions, but these require a collaborative behavior of the people and suitable sensors. Thus, in the case of low resolution, poor views and a non-collaborative public, as in the case with common settings for surveillance cameras, these techniques are not often applicable.

The geometric approaches occur when more than one camera or sensor simultaneously collects information of the same area, and geometric relations among the fields of view (homographies, epipolar lines, and so on) can be adopted to match the data [18,36,37]. The geometric relations, when available, guarantee strong matches or, at least, a stiff candidate selection.

In the general case, only the appearance of the different items can be adopted [38,39]. In the appearance-based approaches, re-id can be correctly done only if the appearance is preserved among the views. It consists of exploiting dress colors and textures, perceived heights and other similar cues and can be considered a soft-biometric approach. Occlusions, illumination changes, different sensor qualities and different viewpoints are some of the challenging issues that make the appearance-based re-id difficult to implement. In [18], Gray et al. for the first time considered the problem of appearance models for person recognition, reacquisition and tracking. Until then, these problems had been evaluated independently, so they called for metrics that apply to complete systems [40,41]. A standard protocol to compare the results is proposed. This is done using the Cumulative Matching Curve (CMC) and introducing the VIPeR dataset for re-id. In [42], an algorithm was proposed that learns a domain-specific similarity function using an ensemble of local features and the AdaBoost classifier. Features are raw color channels in many color spaces and texture information captured by Schmid and Gabor filters [8]. Background clutter highly affects the descriptors of visual appearance for person recognition, and thus, the background modeling is used in many person re-id approaches [38,43,44].

The re-id has even been reinterpreted as a learning problem. In [45], the authors proposed a discriminative model based on the use of Partial Least Squares (PLS). In [46], a robust Mahalanobis metric for Large Margin Nearest Neighbor classification with Rejection (LMNN-R) was obtained with the use of a metric learning framework. Accordingly, in [47], the authors introduced a metric learning approach that learns a Mahalanobis distance from equivalence constraints derived from target labels. A comparison model aimed to maximize the probability of a pair of correctly matched images having a smaller distance than that of an incorrectly matched pair. The model was introduced as the Probabilistic Distance Comparison (PRDC) approach [48]. In [49], the same authors modeled person re-id as a transfer ranking problem, with the main goal of transferring similarity observations from a small gallery to a larger unlabeled probe set. Camera transfer approaches have also been introduced using images of the same person captured from different cameras to learn the associated metrics [50,51]. The Multiple Component Dissimilarity (MCD) framework was defined in [52] to turn a given appearance-based re-id method into a dissimilarity-based one. A supervised technique based on SVM is the approach presented in [53]. Pairs of similar and dissimilar images and a relaxed RankSVM algorithm [54] were used to rank probe images. The main issue with running RankSVM on large datasets is its very expensive computational load due to a large amount of inequality constraints. The authors in [29] used a decision tree to perform a fast matching between descriptors. In this case, the association of the query to one of the models is done by a voting approach. Dimensionality reduction was performed in [30] on image feature vectors through random projection. Afterwards, they built an ensemble of random forests, trained by feature vectors randomly projected onto different subspaces. Random forest was also employed in [31] to learn the similarity function of pairs of person images using color features.

The main differences with our work lay in:

- An RGB-D camera in a top view configuration motivated by the enhancement of the applicability of the proposed approach in crowded public environments is employed. The top-view configuration reduces the problem of occlusions and has the advantage of being privacy preserving because a person’s face is not recorded by the camera [55]. However, this challenging configuration does not allow one to retrieve features related to the front view, which can be highly discriminative for the subject identification. Hence, the proposed approach including the feature extraction and the classification stage was designed according to this challenging setup
- The ensemble classifier was built taking into account the different nature of each feature. The model ensures a higher interpretability with respect to other black box models, allowing one to localize which features contribute to the final prediction.
- The computation time of the training stage is reasonably fast and would be practically feasible for real-world application.

### 2.2. TVPR Dataset and Related Applications

**Figure 1:**
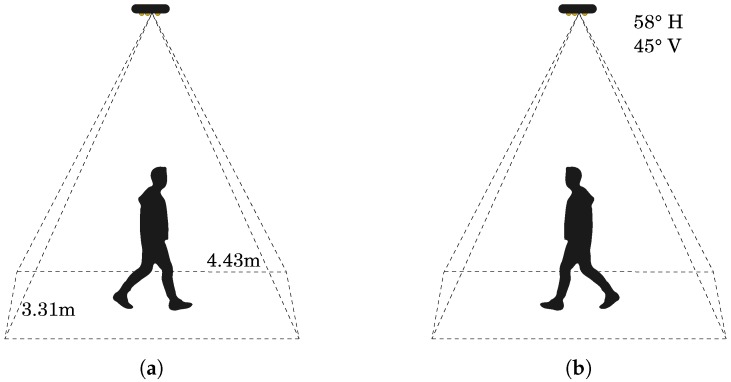
System architecture. (**a**) represents the first passage under the camera as training set, (**b**) is the the returning in the initial position considered as testing set.

TVPR (Top View Person Re-identification) dataset (http://vrai.dii.univpm.it/re-id-dataset) for person re-id [23] contains videos of 100 individuals recorded over several days from an RGB-D camera installed in a top-view configuration. The camera was installed on the ceiling of a laboratory at 4 m above the floor and covered an area of 14.66 m${}^{2}$ (4.43 m × 3.31 m). The camera was positioned above the surface where the analyses took place (Figure 1).

The 100 people of our dataset were acquired in 23 registration sessions. Each of the 23 folders contains a video of one registration session. Acquisitions have been performed over eight days, and the total recording time was about 2000 s.

**Figure 2:**
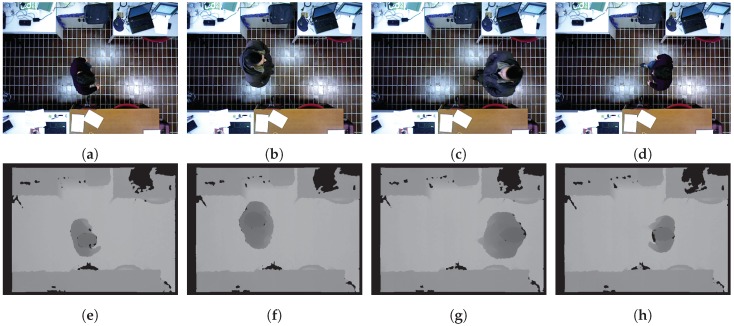
Snapshots of a registration session of the recorded data, in an indoor scenario, with artificial light. People passed under the camera installed on the ceiling. The sequence (**a**–**e**), (**b**–**f**) corresponds to the sequence (**d**–**h**), (**c**–**g**), respectively, training and testing set of the classes 8–9 for the registration session g003.

Registrations were made in an indoor scenario, where people passed under the camera installed on the ceiling. A big issue was environmental illumination. In the recording sessions, the illumination condition was not constant, but it varied as a function of the different hours of the day and also depended on natural illumination due to weather conditions. Snapshots of the video acquisitions, in our scenario, are depicted in Figure 2, where examples of person registration with artificial light are given.

Each person during a registration session walked with an average gait within the recording area in one direction and subsequently turned back and repeated over the same route in the opposite direction. This methodology is used for a better split of the TVPR in the training set (the first passage of the person under the camera) and the testing set (when the person passes a second time under the camera).

Although in the previous datasets presented in the literature, data were gathered using the RGB-D technology, they were not actually suitable for our purposes. The main motivating factors for our top-view dataset are due to some related applications that will be described below.

First, the top-view configuration provides the reliable and occlusion free counting of persons, which is crucial in many applications. Most of the previous works can only count moving people from a single camera, and they fail to count still people or situations when occlusions are very frequent and when there is a crowd. Possible applications can be: safety and security in crowded environments, people flow analysis and access control, as well as counting [56,57,58]. Actual tracking accuracy of top-view cameras overperforms all other tracking methods in crowded environments, with accuracies up to 99%. When there are special security applications or the system is working in usually crowded scenarios, the proposed architecture with the top-view configuration is the only suitable one.

Second, the scope of this specific configuration and analysis is also the interaction detection between people and the environment with the many possible applications for the field of intelligent retail environment such as shopper analytics, in addition to the field of Human Behavior Analysis (HBA) for Ambient Assisted Living (AAL) [59,60,61,62].

Third, another possible application of this specific top-view configuration is fall detection and HBA in smart homes, from high-reliability fall detection to occlusion-free HBA at home for elders in AAL environments [55,63].

All these applications have relevant outcomes from the current research, with the ability to identify users or shoppers while performing tracking, interaction analysis or HBA. Furthermore, all these scenarios can gather data using low-cost sensors and processing units, ensuring scalability and mass usage. Finally, the proposed architecture can be certified on a EU basis privacy by design approach.

## 3. Methodology and Framework

**Figure 3:**
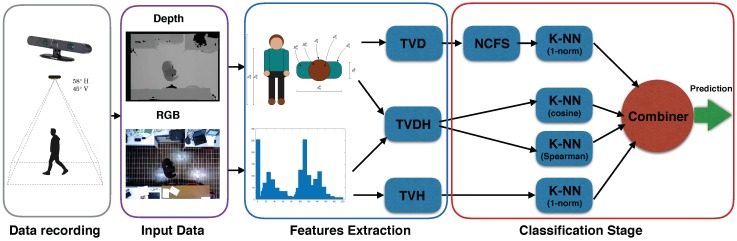
Overview of the proposed approach comprised of data recording, feature extraction and classification stage. NCFS, Neighborhood Component Feature Selection.

Figure 3 shows the overview of the proposed approach comprised of data recording, feature extraction and the classification stage.

### 3.1. Pre-Processing and Feature Extraction

The first step involves the processing of the data acquired from the RGB-D camera. The camera captures the depth and color images, both with dimensions of 640×480 pixels, at a rate up to approximately 30 fps. The scene/objects are illuminated with structured light based on infrared patterns. People were detected from the top-view configuration using the same algorithm employed in [64].

Seven out of the nine features selected are anthropometric features extracted from the depth image: distance between floor and head, $d_{1}$; distance between floor and shoulders, $d_{2}$; area of head surface, $d_{3}$; head circumference, $d_{4}$; shoulder circumference, $d_{5}$; shoulder breadth, $d_{6}$; thoracic anteroposterior depth, $d_{7}$. The remaining two color-based features are acquired by the color image. We also define the color descriptor *TVH*:

(1) $$TVH=\{H_{h}^{p},H_{o}^{p}\}$$

and the depth descriptor *TVD*:

(2) $$TVD=\{d_{1}^{p},d_{2}^{p},d_{3}^{p},d_{4}^{p},d_{5}^{p},d_{6}^{p},d_{7}^{p}\}$$

Finally, *TVDH* is the signature of a person defined as:

(3) $$TVDH=\{d_{1}^{p},d_{2}^{p},d_{3}^{p},d_{4}^{p},d_{5}^{p},d_{6}^{p},d_{7}^{p},H_{h}^{p},H_{o}^{p}\}$$

Color is an important visual attribute for both computer vision and human perception. It is one of the most widely-used visual features in image/video retrieval. To extract these two features, we used HSV histograms. Local histograms have proven to be largely adopted and are very effective. The signature of a person is also composed by two color histograms computed for head/hair and outerwear: $H_{h}^{p}$, $H_{o}^{p}$ in Equation (1), such as in [65], with n=10 bin quantization, for both the *H* channel and *S* channel.

**Figure 4:**
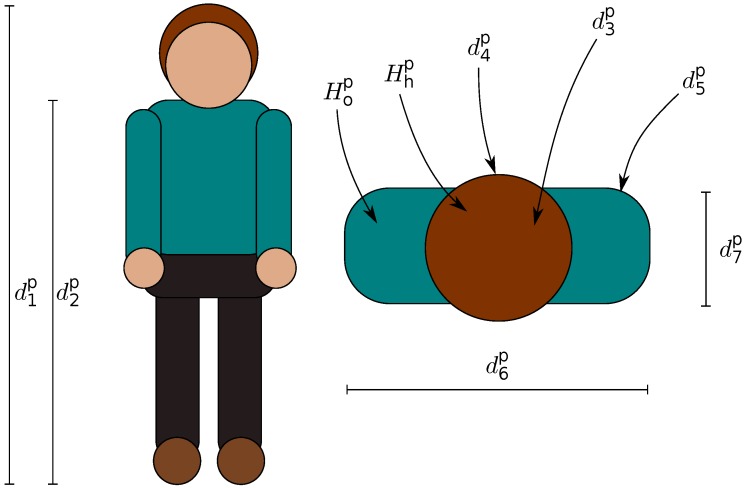
Anthropometric and color-based features.

Figure 4 depicts the set of features considered: anthropometric and color-based.

### 3.2. Classification Stage

The classification stage is depicted in Figure 3. We propose an ensemble classification approach, named Multiple K-Nearest Neighbor (MKNN), where the primary classification stage is represented by different K-NN classifiers according to the nature of the feature descriptors. The overall prediction is performed averaging the computed posterior probability of each K-NN classifier, in order to provide the optimal decision rule.

#### 3.2.1. Predictive Model for TVD Descriptors

Since the TVD descriptors represent anthropometric features, we decided to adopt the 1-norm distance as a discriminative function of the K-NN model and the well-known Neighborhood Component Feature Selection (NCFS) approach [66] in order to learn the optimal feature weighting vector by maximizing the approximate regularized leave-one-out classification error. The application of NCFS allows decreasing the sensitivity of K-NN to irrelevant features [25]. In order to perform feature selection and decrease overfitting, we further introduce the regularization parameter λ, which controls the magnitude of the weighting vector. The optimal lambda found (i.e., $\lambda =5\times 10^{-4}$) was selected by previously implementing a grid-search and optimizing the macro-f1 score in the validation set. For further explanation about NCFS, the reader can refer to [66,67].

#### 3.2.2. Predictive Model for TVH Descriptors

The cosine and the correlation metric are widely used in the literature to measure the similarity among different HSV descriptors [68,69]. Then, we implement two K-NN models with cosine and Spearman rank correlation, respectively, as the distance function.

The cosine distance between two HSV histogram features is defined as:

(4) $$d_{cosine}=1-\frac{TVH_{test_{i}}\cdot TVH_{train_{j}}^{{}^{\prime }}}{∥TVH_{test_{i}}∥∥TVH_{train_{j}}∥}$$

while the Spearman rank correlation-based distance is defined as:

(5) $$d_{spearman}=1-\frac{\left(rg\;TVH_{test_{i}}-rg\;{\bar{TVH}}_{test_{i}}\right)\cdot {\left(rg\;TVH_{train_{j}}-rg\;{\bar{TVH}}_{train_{j}}\right)}^{{}^{\prime }}}{∥(rg\;TVH_{test_{i}}-rg\;{\bar{TVH}}_{test_{i}})∥∥{\left(rg\;TVH_{train_{j}}-rg\;{\bar{TVH}}_{train_{j}}\right)}^{{}^{\prime }}∥}$$

where $TVH_{test}$ and $TVH_{train}$ are converted to ranks $rg\;TVH_{test}$ and $rg\;TVH_{train}$, while $\bar{TVH}$ is the sample mean.

#### 3.2.3. Predictive Model for TVDH Descriptors

For the single K-NN model of the TVDH descriptors, we consider the 1-norm metric, to measure the distance between two different TVDH feature vectors.

### 3.3. Combiner

We introduce the approach for combining the prediction of the single K-NN model. Assuming $\left\{y_{p_{1}},y_{p_{2}},y_{p_{3}},y_{p_{4}}\right\}$ are the predictions of the TVD, TVH and TVDH unseen sample, respectively (i.e., $x_{i}$), if we use the majority vote to determine the final label of $y_{p_{i}}$, the result will be:

(6) $$\underset{y\in 1\ldots 100}{arg max}\sum_{l=1}^{4}\delta \left(y,y_{p_{l}}\right)$$

where δ(a,b)=1 if a=b and δ(a,b)=0 otherwise. The Majority Vote (MV) approach does not take into account the posterior probability and does not always provide the best prediction results. The standard Bayesian approach [70]. finds the most probable hypothesis {y∈1…100} given the observed data $\left\{y_{p_{1}},y_{p_{2}},y_{p_{3}},y_{p_{4}}\right\}$:

(7) $$\underset{y}{arg max}P\left(y|\left\{y_{p_{1}},y_{p_{2}},y_{p_{3}},y_{p_{4}}\right\}\right)$$

according to Bayes’ theorem, the maximally probable hypothesis becomes:

(8) $$\underset{y}{arg max}P\left(\left\{y_{p_{1}},y_{p_{2}},y_{p_{3}},y_{p_{4}}\right\}|y\right)P\left(y\right)$$

The Bayesian approach selects the model with the highest posterior probability and then proceeds as if the selected model had generated the data.

Differently from the Bayesian approach, we compute the average of the posterior probability (i.e., $P(\bar{y}))$ of the 4 hypotheses as follows:

(9) $$P\left(\bar{y}\right)=\sum_{l=1}^{4}P\left(y|y_{p_{l}}\right)=\sum_{l=1}^{4}P\left(y_{p_{l}}|y\right)P\left(y\right)$$

and the final prediction is:

(10) $$y_{p}=\underset{\bar{y}\in 1\ldots 100}{arg max}P\left(\bar{y}\right)$$

Our ensemble methodology is based on Bayesian Model Averaging (BMA), which is an application of Bayesian inference to the problems of combined prediction of different classifiers. Although this choice can lead to overfitting in some situations [71], it provides straightforward model choice criteria and less risky predictions [72,73,74]. The BMA ignores the uncertainty in model selection, leading to over-confident inferences and decisions [73].

## 4. Results

The baseline results are reported in Section 4.1 in terms of the Cumulative Match Curve (CMC). In Section 4.2 and Section 4.3, however, we show the results of the proposed MKNN approach for re-id classification. The authors compare the performance of the proposed methodology with respect to single K-NN classifiers and other supervised machine learning algorithms widely used in the re-id literature. We have also performed the computation time comparison related to the training stage.

### 4.1. Baseline Results

**Figure 5:**
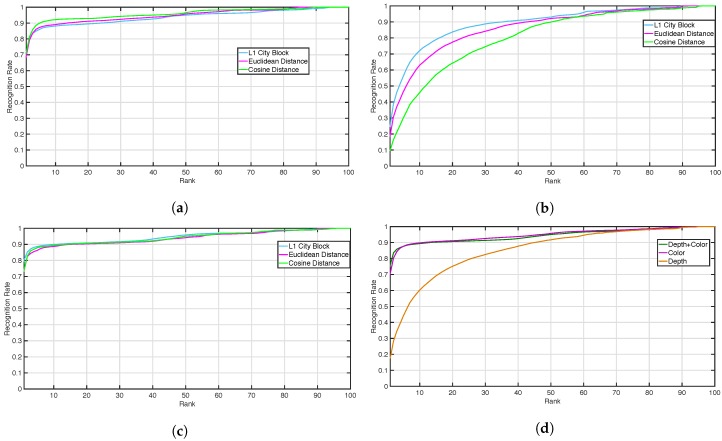
The baseline Cumulative Matching Curve (CMC) curves obtained on the Top View Person Re-Identification (TVPR) dataset. (**a**,**b**) shows respectively the CMC obtained using the *TVH* and *TVD* descriptors for three different distance: one-norm (L1 city block, cyan), two-norm (euclidean, purple) and cosine (green). (**c**) provides the CMC computed using both the *TVH* and *TVD* descriptors (i.e., *TVDH*), while (**d**) is the averaged CMC over the three considered distance for the color (i.e., average of CMC curves in (a), purple), depth (i.e., average of CMC curves in (b), orange) and depth + color (i.e., average of CMC curves in (c), green).

The baseline performance of the TVPR dataset was evaluated in terms of recognition rate, using the CMC curves, as previously described in [23]. Figure 5 depicts a comparison among the *TVH*, *TVD* and *TVDH* predictors in terms of CMC curves, to compare the ranks returned by using these different descriptors, where the horizontal axis is the rank of the matching score and the vertical axis is the probability of correct identification.

In particular, Figure 5a,b represents respectively the CMC obtained using the *TVH* and *TVD* descriptors for three different distances: one-norm (L1 city block), two-norm (euclidean) and cosine. Figure 5c provides the CMC computed using both *TVH* and *TVD* descriptors (i.e., *TVDH*), while Figure 5d is the averaged CMC over the three considered distances for the color (i.e., average of CMC curves in Figure 5a), depth (i.e., average of CMC curves in Figure 5b) and depth + color (i.e., average of CMC curves in Figure 5c). Although it can be assumed that the best performance was achieved when using the combination of descriptors (*TVDH*), the contribution of the depth was small, and the CMC curves in Figure 5a,c are very similar. However, the depth information can be informative for the re-id task (see Figure 5b). These baseline results suggest the need for a methodology to combine the different nature of descriptors, exploiting the importance and potential of the depth information. In this context, our approach aimed to exploit the informative power of depth and RGB input, properly combining the different nature of each feature.

### 4.2. Results of the Proposed Approach

We considered the first passage under the camera as the training set and the return to the initial position as the testing set. The dataset was composed of 21,685 instances divided into 11,683 for training and 10,002 for testing. The performance of the proposed MKNN method is reported in Table 1 in terms of macro-F1 score, precision and recall. We also report the results of the single K-NN classifier for each descriptor (i.e., TVH, TVD, TVDH) and each different distance (i.e., cosine, Spearman’s rank correlation and one-norm). We have highlighted in bold the single K-NN used for designing the proposed MKNN method. The optimal number of neighbors is five, and it has been chosen since it maximizes the macro-F1 score in the validation set. Additionally, we have reported the results of different combiner approaches (i.e., MV, Bayesian and BMA). The proposed BMA-MKNN approach performed favorably over the other methods.

**Figure 6:**
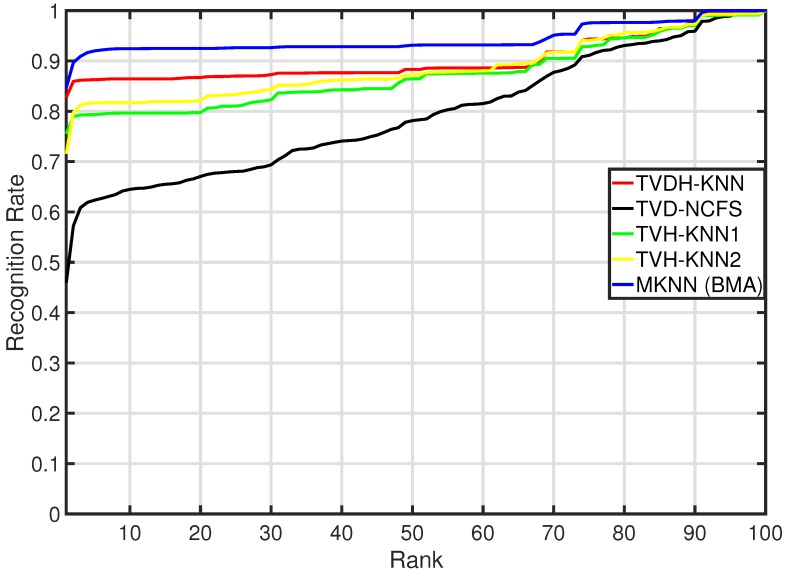
The CMC curves of the MKNN and the standard K-NN methods.

According to the nature of the descriptors, the cosine distance was the most consistent measure in order to achieve the best performance for the TVH input, while the K-NN with one-norm achieved the best performance considering the TVDH input. The proposed MKNN methodology outperformed all single K-NN classifiers. In particular, the MKNN improved the performance of TVD-KNN, TVH-KNN and TVDH-KNN by 84.44%, 12% and 2.5%, respectively. Figure 6 shows the CMC curve of the MKNN compared with respect to the CMC curves of the single weak learner fed with TVH, TVD and TVDH. The ranking returned by MKNN showed better performance than the single classifier. This result outlines the advantage of the proposed approach in order to exploit the discriminative power of the depth information for the re-id task. In addition, the introduced BMA approach performed favorably over the MV and Bayesian methods.

**Figure 7:**
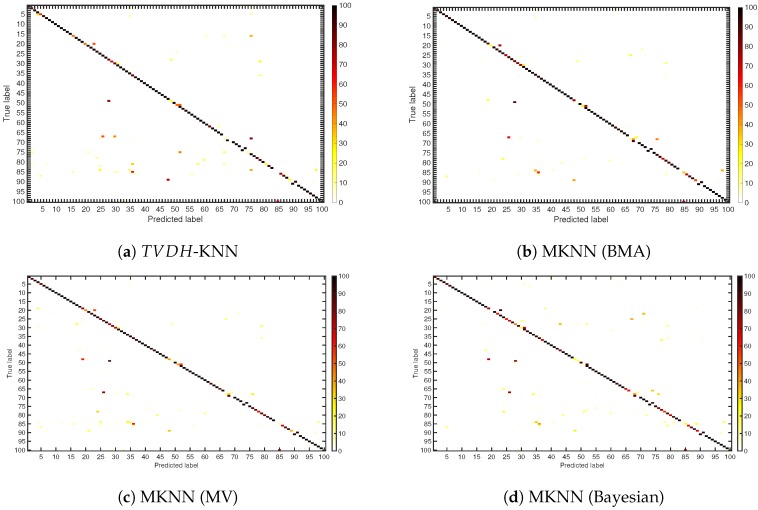
Confusion matrices of TVDH-KNN, MKNN (BMA), MKNN (MV) and MKNN (Bayesian).

In order to highlight the misclassification error, we disclose in Figure 7 the confusion matrices of the TVDH-KNN, MKNN (BMA), MKNN (MV) and MKNN (Bayesian). The MKNN (BMA) shows a lower number of misclassified id-subject with respect to TVDH-HNN, MKNN (MV) and MKNN (Bayesian).

**Figure 8:**
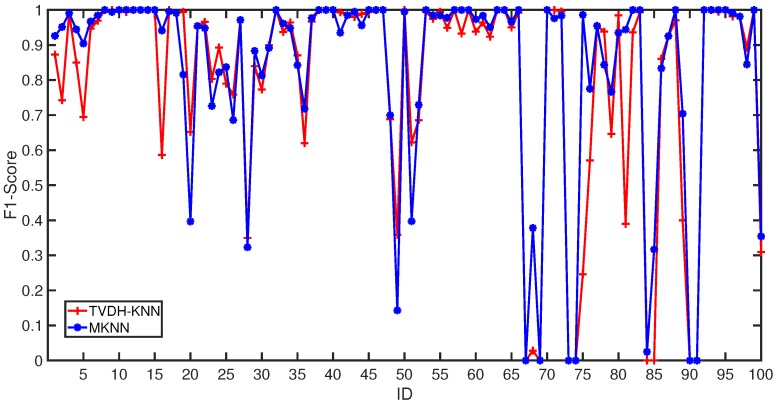
The macro-F1 for each subject for the MKNN and standard K-NN method.

We summarize in Figure 8 the macro-f1 score for the MKNN and the TVDH-KNN for each class (subjects). The macro-f1 score is the same for 32 out of 100 subjects, while the MKNN achieves higher performance than TVDH-KNN in 42 out of 100 subjects. This result suggests how the MKNN (BMA) recognizes 10% of subjects with a higher recognition rate with respect to TVDH-KNN.

**Figure 9:**
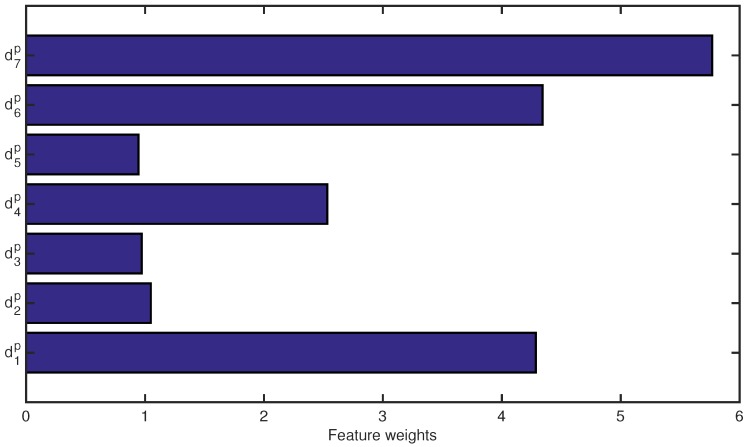
The optimal feature weights for TVD descriptors found by the NCFS algorithm.

The implemented NCFS for the TVD descriptors allowed decreasing the generalization error of the standard K-NN classifier while increasing the sparsity, as well as the interpretability of the model. Moreover, also the increase of K-NN performance in terms of precision, recall and macro-f1 score can be seen in Table 1. The optimal weighting vector found by the NCFS algorithm is shown in Figure 9. The feature with the highest predictive power is the thoracic anteroposterior depth ($d_{7}$), while the less relevant TVD descriptors are the distance between floor and shoulders ($d_{2}$), the area of the head surface ($d_{3}$) and the shoulder circumference ($d_{5}$).

### 4.3. Comparison with the Standard Supervised Machine Learning Algorithm

Table 2 shows the comparison between our approach and standard supervised learning algorithms widely adopted in the re-id scenario such as DT [29], bagged tree, RF [30,31], adaptive boosting (AdaBoost), linear programming boosting (LPBoost) and totally corrective boosting (TotalBoost). The considered inputs for the DT, bagged tree, RF, AdaBoost, LPBoost and TotalBoost classifiers are the TVDH descriptors.

The MKNN outperformed all standard methods, achieving an improvement of 76.60%, 3.75%, 18.57%, 43.10%, 69.39% and 36.07% with respect to DT, bagged tree, RF, AdaBoost, LPBoost and TotalBoost. The K-NN may perform better than DT and RF when the number of training samples is not huge compared to the number of classes. The advantage of our ensemble strategy lies in the way we have built and combined each classifier. In particular, each weak learner was built according to the different nature of the features in order to extract the discriminative information of each subject. Differently from our approach, the other boosting and bagged strategies combined different weak learners in an automatic fashion without taking into account the different descriptors (i.e., TVH and TVD).

Table 3 shows the computation time expressed in seconds (s) for the training stage of all methodologies. MKNN (BMA) was reasonably fast and would be practically feasible for the re-id task.

## 5. Conclusions and Future Works

In this paper, we describe a method for person re-identification based on features derived from both depth (anthropometric features) and color. Different from other approaches, the experiments were conducted on the TVPR dataset where the RGB-D images were collected in a top-view setting, reducing the problems of occlusions, while preserving the privacy issue [55].

Person recognition is handled by using the proposed ensemble method, named Multiple K-Nearest Neighbor (MKNN), based on the combination of different K-NN classifiers. Each K-NN is built with a different distance function based on the nature of the feature descriptors, and the neighborhood component feature selection is introduced for the anthropometric features. The experimental results demonstrate how the proposed methodology outperforms standard supervised classifiers (i.e., k-NN, DT, bagged tree, RF and boosting methods). Moreover, the computation time analysis of the training stage suggests that the proposed MKNN method is reasonably fast, encouraging the application of the proposed approach for the person re-identification task in the retail scenario. This improvement may be explained by the fact that our approach is consistent to model and combine the nature and information of different descriptors (i.e., TVH and TVD), weighting the importance of the anthropometric features. Further investigation will be devoted to improve our approach by extracting other informative features and setting up the proposed approach for the real-time processing of video images in the retail scenario. In the field of retail applications, the long-term goal of this work is to merge the developed re-identification system with an audio framework and the use of other types of RGB-D cameras, such as Time Of Flight (TOF) ones. The system can be integrated additionally as a source of high semantic level information in a networked ambient intelligence scenario, to provide cues for different problems, such as detecting abnormal speed and dimension outliers, alerting one to a possible uncontrolled circumstance. It would also be interesting to evaluate both color and depth images in a way that it does not decrease the performance of the system when the color image is being affected by changes in pose and/or illumination. 

## Figures and Tables

**Table 1:** Classification results for single K-NN and Multiple K-Nearest Neighbor (MKNN) algorithms. BMA, Bayesian Model Averaging.

	Classifier	Distance	Precision	Recall	Macro-F1 Score
**TVD**	**KNN + NCFS**	1-norm	0.49	0.46	0.45
	KNN	1-norm	0.38	0.36	0.34
**TVH**	**KNN**	cosine	0.77	0.76	0.74
	**KNN**	Spearman	0.75	0.73	0.71
	KNN	1-norm	0.76	0.76	0.74
**TVDH**	**KNN**	1-norm	0.83	0.82	0.81
	KNN	2-norm	0.81	0.80	0.78
	**MKNN (MV)**		0.83	0.83	0.81
	**MKNN (Bayesian)**		0.81	0.80	0.78
	**MKNN (BMA)**		**0.86**	**0.85**	**0.83**

**Table 2:** Comparison of MKNN with respect to the standard supervised learning approach. LPBoost, linear programming boosting.

Classifier	Input	Precision	Recall	F1-Score
KNN	TVDH	0.83	0.82	0.81
DT	TVDH	0.52	0.50	0.47
Bagged Tree	TVDH	0.83	0.81	0.80
RF	TVDH	0.74	0.72	0.70
AdaBoost	TVDH	0.65	0.60	0.58
LPBoost	TVDH	0.57	0.52	0.49
TotalBoost	TVDH	0.69	0.62	0.61
**MKNN (BMA)**		**0.86**	**0.85**	**0.83**

**Table 3:** Computation time training stage.

Classifier	Training Time (s)
KNN	0.02
DT	1.31
Bagged Tree	12.14
RF	113.21
AdaBoost	31.14
LPBoost	375.94
TotalBoost	576.24
**MKNN (BMA)**	6.94
